# Drug-resistant Epilepsy: Which Drugs are Substrates of P-glycoprotein and Which are Not?

**DOI:** 10.2174/011570159X381818250612061802

**Published:** 2025-06-30

**Authors:** Javier Aylon Val, Virgilio Hernando-Requejo

**Affiliations:** 1 Department of Internal Medicine, Severo Ochoa University Hospital, Leganés, Madrid, Spain;; 2 Department of Neurology, Severo Ochoa University Hospital, Leganés, Madrid, Spain;; 3 Department of Neurology, HM Sanchinarro University Hospital, Madrid, Spain;; 4 Faculty of Medicine, San Pablo CEU University, Alcorcón, Madrid, Spain

**Keywords:** P-Glycoprotein, antiseizure medication, nervous system, gene knockout, antiepileptic drugs, drug-resistant epilepsy

## Abstract

One of the accepted factors of antiseizure medication resistance is the action of P-glycoprotein (P-gp), limiting the access of drugs to the nervous system. But if we ask which antiseizure medications are substrates of P-gp and which are not, the available bibliography will not allow us to obtain a clear answer. In this review, we focus on clarifying this response. The reviewed studies have been conducted both in cell lines and in mice that have been administered a P-gp inhibitor, artificially induced with drug-resistant epilepsy, or had a P-gp gene knockout. A limited number of studies have been conducted in dogs, primates, brain sections of known epilepsies, or human volunteers, including pharmacokinetic studies in healthy volunteers and symptomatic response to treatment. Notably, in human cases, allele variation studies check if having one allele or another of P-gp varies the pharmacokinetics in question. As we see, the approach to P-gp and antiseizure medication can be done using very different methods, which undoubtedly complicates the interpretation of the findings. We cannot be categorical in our results, but we can mention probabilities. Regarding the weighting of studies, we will consider those conducted in humans as more important, followed by animal studies, and we will give less weight to studies showing contradictory results compared to the general bibliographic base. Based on the published bibliography, we propose that, among the anti-crisis medications, the following are likely substrates of P-glycoprotein: Phenytoin, Phenobarbital, Oxcarbazepine, Lamotrigine, Topiramate, and Lacosamide (less evidence). The following are probably not substrates: Brivaracetam, Zonisamide, Valproic acid, Perampanel, Gabapentin, and Vigabatrin. We have not obtained enough information about: Carbamazepine, Eslicarbazepine, Levetiracetam, Tiagabine, Felbamate, Pregabalin, Rufinamide, Ezogabine, and Retigabine.

## 1 INTRODUCTION

The medical treatment of epilepsy controls most patients; with the first drug tested, we can expect that 50% will be seizure-free. This contrasts with the fact that one-third of patients will not be controlled, despite having several drugs with different mechanisms of action. Drug resistance is defined as the failure of adequate trials of two tolerated and appropriately chosen and used antiseizure medication (ASM) schedules (whether as monotherapies or in combination). Why this drug resistance occurs is not clear; it is considered that different factors are involved, one of which is probably the restriction of drug entry into the central nervous system by the action of P-glycoprotein (P-gp) [1]. If this is truly a factor of drug resistance, it would be useful to know which antiseizure medication (ASM) are substrates of P-gp and which are not, but the bibliographic sources do not clarify this information, as they provide contradictory results, do not include the most modern drugs, or make a review that, due to its exhaustiveness, makes it difficult to answer this specific question. In this review, we analyze the available literature to characterize ASM as substrates or not of P-gp.

## 2 MATERIALS AND METHODS

We conducted a bibliographic search as follows:

- In PubMed: first, we paired each antiepileptic drug with “p-glycoprotein”, then “antiepileptic” and “p-glycoprotein”.
- For some drugs, we did not obtain published information; therefore, we searched for references from the pharmaceutical industry or regulatory agencies regarding them.
- The ASM included in this review are: phenytoin, phenobarbital, carbamazepine, oxcarbazepine, eslicarbazepine, lamotrigine, lacosamide, topiramate, levetiracetam, tiagabine, felbamate, brivaracetam, zonisamide, vigabatrin, valproic acid, perampanel, gabapentin, pregabalin, rufinamide, ezogabine, and retigabine. Benzodiazepines were not included due to the lack of basic studies considering them (the available information was taken from Google and was not rigorous).

## 3 RESULTS AND DISCUSSION

We have found 83 references that provide useful information to answer the question: Which ASMs are substrates of P-gp and which are not? We use the term 'positive' for drugs that are substrates of P-gp, and 'negative' for those that are not.

Some general considerations about the results obtained:

- The reviewed studies have been conducted both in cell lines and in mice that have been administered a P-gp inhibitor, artificially induced with drug-resistant epilepsy, or had a P-gp gene knockout. A limited number of studies have been conducted in dogs, primates, brain sections of known epilepsies, or human volunteers, including pharmacokinetic studies in healthy volunteers and symptomatic response to treatment. Notably, in human cases, allele variation studies check if having one allele or another of P-gp varies the pharmacokinetics in question.
- Regarding false negatives in cell lines, antiseizure medications diffuse by osmosis very easily, so they can often experience false negatives. This likely explains the negativity of some studies in cell lines of phenobarbital and phenytoin.
- Regarding allele studies, negativity does not provide information in one way or another, since the variability of an allele does not necessarily affect the transport of a drug even if it is a substrate. However, we find positivity useful as it confirms the interaction of the drug as a substrate with P-gp.
- Regarding the weighting of studies, we will consider those conducted in humans (if they conclude that the ASM under study is a substrate) as more important, followed by animal studies, and we will give less weight to studies showing contradictory results compared to the general bibliographic base.
- We are aware of the existence of high-quality studies that we did not mention, such as comprehensive reviews or data from computational, bioinformatics, and pharmacogenetics insights [2-4], but we preferred to simplify the search by focusing on the medications. Likewise, we must point out that, also to answer precisely the question that makes up the title of this article, we have limited the analysis to trying to clarify specifically whether or not anti-seizure drugs are substrates of P-gp, even though we know that the pharmacological relationship between drugs and this protein can be broader.

Results of the review for each drug:

- Phenytoin is considered a substrate by the vast majority of studies. The studies that confirm it as a substrate are [1, 5-30]. The only exceptions are [31-35], with the last three being cell line studies and study [35] acknowledging that its results do not match previous evidence; 26 studies against five.
- Phenobarbital is considered a substrate by all studies [1, 14, 34, 36-46], except in [33, 35, 47]; 14 studies against three.
- From the carbamazepine family, we would like to propose oxcarbazepine as a P-gp substrate, given that it has the largest body of evidence, including in humans in a study [48]. The studies that recognize it as a substrate are [23, 32, 48-51]; 6 studies against zero. Eslicarbazepine is considered a substrate in study [49], the only study that considers it, while carbamazepine varies from study to study, being positive in [1, 15, 21, 23, 35, 52-56], variable in [32], and negative in [19, 34, 57-61]. The explanation given by the study [62] is that its metabolites are substrates, but carbamazepine itself is not.
- Lamotrigine presents negative studies [23, 33, 47] and positive studies [1, 28, 34, 35, 38, 63, 64], with the latter being more numerous, seven studies against three. Regarding the studies that do not consider it a substrate, studies [33] and [47] consider other ASMs as non-substrates against previously known scientific evidence, and study [23] was conducted in cell lines.
- Lacosamide is considered a substrate by the two studies found, [65] and [66].
- Topiramate is considered a substrate by studies [13, 67] and [35], and not a substrate by other studies [1, 33] and [34]. All the studies that do not consider it a substrate make errors with other antiseizure medications and are in cell lines, while two of the three studies that consider it a substrate are in animals, and study [35] which acknowledges its limitations in determining substrates for other antiseizure medications, refers to the clear positivity of topiramate. It is also considered a substrate by DrugBank.
- The evidence on levetiracetam varies, being positive in studies [16, 33, 54, 68, 69], and negative in studies [29, 47, 68]. Study [47] is a cell line study, and both studies [70] and [29] contradict the scientific evidence regarding other antiseizure medications. Molecular study [71] considers it a definitive substrate but with lower affinity than other substrates. There is a scientific consensus on its nature as a substrate.
- Tiagabine is considered a substrate by the only study that takes it into account [13].
- Felbamate is considered a substrate by study [38] and not a substrate by study [34].
- Brivaracetam is considered a non-substrate by the two studies that consider it [71, 72], and in its technical data sheet, although DrugBank considers it a substrate. Given the scientific evidence, maybe this is an error, and it is one of the drugs we can most firmly consider a non-substrate.
- Zonisamide is considered a non-substrate by the two studies that consider it [34, 73], and study [70] in fact uses it as a negative control.
- Vigabatrin is considered a non-substrate by the three studies that consider it [1, 33, 35]. We understand that this drug is less useful than others in clinical practice.
- Regarding valproic acid, some studies consider it a substrate [1, 64, 74], with [1] and [74] being cell line studies, and study [1] containing information contrary to the scientific evidence regarding other antiseizure medications. However, most of the evidence, which is very abundant for this drug, does not consider it a substrate. Independent studies that do not consider it a substrate include [47, 54, 59-61, 75] ([54] is allele-negative), and [76]. There is an extensive review of multiple studies in cell lines, live animals, and animal brains from 2007 referenced in [77], where it is unanimously not considered a substrate.
- Perampanel is considered a non-substrate in its technical data sheet, apparently after *in vitro* studies referenced in [78] and [69], although we have not located the primary studies. There is no evidence that considers it a substrate, and DrugBank considers it a non-substrate, apparently following FDA guidelines outlined in [79].
- Gabapentin is considered a non-substrate by studies [1, 33] and [35], with [80] and [81] being negative allele studies, as well as in its technical data sheet and DrugBank, and indirect references in [82] and [83]. Only study [34], a cell line study, considers it a weak positive.
- Pregabalin is considered a non-substrate by study [73].
- Rufinamide is considered a non-substrate by the only study that takes it into account [73].
- Ezogabine and retigabine are considered non-substrates in their technical data sheet, apparently in the context of a personal communication.

## CONCLUSION

The approach to P-gp and ASM, which are substrates of the same, can be done using very different methods, which undoubtedly complicates the interpretation of the findings. In addition to this, we have found contradictory results, not always attributable to studies that “went against the grain”. Both in cell line studies and in humans, false negatives are considered, which is why we have given special importance to positive results. These limitations prevent us from providing categorical results, but we can provide probabilities (Table **1**). We consider the following drugs as probable P-gp substrates: phenytoin, phenobarbital, oxcarbazepine, lamotrigine, topiramate, and, to a lesser extent, lacosamide. In all cases, we can rely on animal studies, and in all except topiramate, on human studies.

As drugs that are probably not P-gp substrates, we propose: brivaracetam, zonisamide, valproic acid, perampanel, gabapentin, and vigabatrin.

The available bibliography, due to its scarcity or contradictory nature, does not allow us to position the rest of the studied ASM: carbamazepine, eslicarbazepine, levetiracetam, tiagabine, felbamate, pregabalin, rufinamide, ezogabine, and retigabine.

Given that P-gp activity is a recognized factor in drug resistance, we believe that the information provided can help analyze the extent of its influence and facilitate the design of clinical studies that assess this resistance factor when establishing AMS in monotherapy or polytherapy.

## AUTHORS’ CONTRIBUTIONS

The authors confirm their contribution to the paper as follows: study concept or design: VHR; writing the paper: JAV. All authors reviewed the results and approved the final version of the manuscript.

## Figures and Tables

**Table 1:** Anti-seizure medications, probability of being or not substrates of P-gp.

Probable P-gp substrates	Phenytoin
	Phenobarbital
	Oxcarbazepine
	Lamotrigine
	Topiramate
	Lacosamide*
Probably not P-gp substrates	Brivaracetam
	Zonisamide
	Valproic Acid
	Perampanel
	Gabapentin
	Vigabatrin
Inconclusive information	Carbamazepine Eslicarbazepine Levetiracetam Tiagabine Felbamate Pregabalin Rufinamide Ezogabine Retigabine

**Note:** *Lacosamide: less evidence.

## LIST OF ABBREVIATIONS

ASM: Antiseizure Medication
P-gp: P-glycoprotein
